# Factors affecting the documentation of spoken safety-netting advice in routine GP consultations: a cross-sectional study

**DOI:** 10.3399/BJGP.2021.0195

**Published:** 2021-09-07

**Authors:** Peter J Edwards, Ian Bennett-Britton, Matthew J Ridd, Matthew Booker, Rebecca K Barnes

**Affiliations:** Centre for Academic Primary Care, Bristol Medical School, University of Bristol, Bristol.; Centre for Academic Primary Care, Bristol Medical School, University of Bristol, Bristol.; Centre for Academic Primary Care, Bristol Medical School, University of Bristol, Bristol.; Centre for Academic Primary Care, Bristol Medical School, University of Bristol, Bristol.; Centre for Academic Primary Care, Bristol Medical School, University of Bristol, Bristol; senior qualitative researcher, Nuffield Department of Primary Care Health Sciences, University of Oxford, Oxford.

**Keywords:** health communication, medical records, medicolegal, patient safety, primary care, safety-netting

## Abstract

**Background:**

Previous studies have reported how often safety-netting is documented in medical records, but it is not known how this compares with what is verbalised and what factors might influence the consistency of documentation.

**Aim:**

To compare spoken and documented safety-netting advice and to explore factors associated with documentation.

**Design and setting:**

A cross-sectional study, using an existing GP consultations archive.

**Method:**

Observational coding involving classifying and quantifying medical record entries and comparison with spoken safety-netting advice in 295 video-/audio-recorded consultations. Associations were tested using logistic regression.

**Results:**

Two-thirds of consultations (192/295) contained spoken safety-netting advice that applied to less than half of the problems assessed (242/516). Only one-third of consultations (94/295) had documented safety-netting advice, which covered 20.3% of problems (105/516). The practice of GPs varied widely, from those that did not document their safety-netting advice to those that nearly always did so (86.7%). GPs were more likely to document their safety-netting advice for new problems (*P* = 0.030), when only a single problem was discussed in a consultation (*P* = 0.040), and when they gave specific rather than generic safety-netting advice (*P* = 0.007). In consultations where multiple problems were assessed (*n* = 139), the frequency of spoken and documented safety-netting advice decreased the later a problem was assessed.

**Conclusion:**

GPs frequently do not document the safety-netting advice they have given to patients, which may have medicolegal implications in the event of an untoward incident. GPs should consider how safely they can assess and document more than one problem in a single consultation and this risk should be shared with patients to help manage expectations.

## INTRODUCTION

Safety netting is a broad concept that has been used to describe a diverse array of clinical activities for managing ‘what if?’ scenarios and clinical uncertainty.^1^^–^2 The primary focus of safety-netting is appraising ‘what could go wrong?’ and ‘how can I keep this patient safe?’. More specifically, but often used synonymously, ‘safety-netting advice’ is defined as: ‘ *Information shared with a patient or their carer designed to help them identify the need to seek further medical help if their condition fails to improve, changes, or if they have concerns about their health*’.^3^

Since its original description by Neighbour,^4^ safety-netting has been widely advocated by many professional bodies and included in guidelines and clinical standards.^5^^–^8 Subsequently, clinicians have been reprimanded for its omission or inadequate documentation.^9^ There is broad consensus among clinicians that safety-netting advice should be recorded in patients’ medical records.^10^ This is important not only for safe handover between clinicians at a time when continuity of care can be limited,^11^ but also from a medicolegal perspective.^12^ However, research to date suggests safety-netting documentation is often absent or incomplete and there is often discordance between patients’ reports of consultations and medical records.^13^^–^15 Understanding the gap between what is said and what is documented, and the factors that contribute to this, may help protect both clinicians and patients from harm.

Previous studies have assessed the binary presence or absence of safety netting in medical records from UK GP consultations^14^^,^^16^ and reported on discrepancies between independent review of recorded consultations and electronic health records in the UK and US.^15^^,^^17^ This study builds on a previous study (by the same author group) that detailed analysis of spoken safety-netting advice in routine GP consultations^14^ and presents a framework for assessing safety-netting advice in medical records that can be used to audit local practice.

The aim of the study was to evaluate how spoken safety-netting advice compared with what was recorded in the medical records in routine GP consultations, and explore factors that may have influenced GPs’ documentation practices. This information can then be used to inform clinician training and practice.

## METHOD

### Participants

The data used in this secondary analysis were obtained from the ‘One in a Million’ Primary Care Consultations Archive, details about the archive are reported elsewhere.^18^^–^19 In brief, adult patients (aged ≥18 years) attending for routine face-to-face appointments with 23 participating GPs from 12 general practices in the West of England in 2014–2015 were approached to have their consultation video- or audio-recorded, and the corresponding medical record entries for that consultation and return visits for the same problem in the following 3 months collected. This study includes data from patients who consented for their data to be re-used by the original research team and where medical records were available (295 of 327 patients).

**Table:** How this fits in

Previous research has provided qualitative insights into how GPs document safety-netting advice and there have been quantitative reports of the binary presence or absence of safety-netting in medical records. To the authors’ knowledge, this is the first study to undertake a detailed analysis of the content of documented safety-netting advice and make objective comparisons with what was spoken in recorded consultations. GPs more frequently documented their safety-netting advice if it was specific (for example, *‘I’d want you to come back if you start coughing up horrid coloured stuff, greeny-browny, or if you start coughing up any blood, or if you feel more short of breath’*) rather than generic advice (for example,, *‘any problems let me know’*), for a new problem, and for problems that were the entire focus of a consultation.These trends in GP documentation practices highlight that certain consultations, such as those where multiple problems are assessed, may represent a higher medicolegal risk to GPs because of incomplete documentation, and these potential biases should be considered in medical records based research.

### Content of consultations

The characteristics of problems discussed in all consultations had already been coded by direct observation of video-recordings and review of verbatim transcripts.^14^^,^^19^ Coding is updated if discrepancies are identified in additional projects using the archive. Problems were defined as the answer to the question ‘what is wrong?’.^20^

### Coding

Two coders independently screened a random 10% (30 consultations) of the medical record entries for evidence of safety-netting advice. A modified version of an Excel-based Safety-Netting Coding Tool (SaNCoT)^3^ was developed for use on medical records and the relevant text from all consultations were entered into the tool (SaNCoT medical notes edition 1.1, see Supplementary Table S1).^21^ Eleven problems — 10% of all problems that included documented safety-netting advice — were selected at random and coded independently by the same two authors. Inter-rater reliability (IRR) scores were assessed, after which the first author coded the remaining consultations.

### Software and statistical analysis

Data were exported into Stata 16.1 for analysis. IRR scores were generated using Cohen’s κ with quadratic weighting for continuous variables.^22^^–^23 Univariable and multivariable logistic regression models were generated to explore factors that may have affected the decision made by GPs about whether to document safety-netting advice they had verbalised or problems they had assessed. Multilevel mixed-effects models were used to adjust for clusters of problems seen by the same GP or multiple problems raised by the same patient in all models. Wald tests were used to assess the hypotheses that Index of Multiple Deprivation (IMD) quintile was associated with an altered frequency of safety-netting advice in the adjusted models. Odds ratios (OR) are reported using a significance level of 0.05 with 95% confidence intervals (CIs). Multivariable models excluded variables with missing data.

A subgroup analysis of the previously described multivariable logistic regression model was repeated with minor amendments.^14^ In this analysis, variables associated with a higher or lower frequency of safety-netting advice were assessed and models of spoken and documented safety-netting advice were compared. In the model assessing documented safety-netting advice, problems that were verbally discussed but not documented were excluded and the code ‘was this the only problem assessed during the consultation?’ was updated to reflect only documented problems. Data from consultations with missing data were excluded.

## RESULTS

### Participant characteristics

A summary of GP and patient details is provided in Table 1. There were more female patients (63.7%) and female GPs (56.5%). Most patients (87.5%) and all GPs self-reported white ethnicity. The mean average patient age was 50.9 years (standard deviation [SD] 19.3, range 18–96 years), and the average GP age was 45.6 years (SD 8.7 range 32–62 years).

**Table 1.** Characteristics of patients and GPs

**Group**	***n* (%)**
**Patients (*n*= 295)**	
Patient sex	
Male	107 (36.3)
Female	188 (63.7)
Patient age, years	
18–34	76 (25.8)
35–49	51 (17.3)
50–64	74 (25.1)
≥65	81 (27.5)
Not reported	13 (4.4)
Patient ethnic group	
White	258 (87.5)
Other	29 (9.8)
Not reported	8 (2.7)
Index of Multiple Deprivation quintile	
1st (least deprived)	95 (32.2)
2nd	48 (16.3)
3rd	31 (10.5)
4th	46 (15.6)
5th (most deprived)	74 (25.1)
Not reported	1 (0.3)

**GPs (*n*** **= 23)^a^**	
Doctor sex	
Male	10
Female	13
Doctor age, years	
18–34	3
35–49	11
50–64	9
Doctor ethnic group	
White	23
Other	0
Not reported	0
Doctor role	
Partner	19
Salaried	4

a *Given the sample size for GPs of 23, percentages are not given.*

### IRR

When given three options for each verbally discussed problem: safety-netting advice present, absent, or problem not documented, IRR agreement was 47/49 (95.9%, κ = 0.92). The IRR agreement for the presence or absence of documented safety-netting advice in each consultation was 29/30 (96.7%) κ = 0.89. The mean average agreement for the application of the safety-netting coding tool was 96.1% (κ = 0.87, Table 2).

**Table 2.** Inter-rater reliability of coding tool^a^

**Code**	**Inter-rater reliability score**	

	**%**	κ **(weighted)**
1.1 Problem or treatment safety-netting advice	81.8	0.54
2.1 Format	100	^c^
3.1 Number of conditions/symptoms^b^	100	1 (1)
3.2 Free-text conditions/symptoms	–	–
3.3 Generic or specific symptoms/conditions	90.9	0.81
4.1 Action advised	100	^c^
4.2 Timescale of action	100	1
5.1 Communication/written advice	100	1
Average	96.1	0.87

a *Results produced from two coders’ independent review of 11 problems in 11 consultations.*

b *Code 3.1: quadratic weighting used for continuous variables.*

c *Incalculable as no variability in data, for example, all conditional + course of action format reported by both coders.*

### Comparison between spoken and documented safety-netting advice

Verbalised safety-netting advice was given in 65.1% (192/295) of consultations and for 46.9% (242/516) of problems. However, where safety-netting advice was given verbally, it was only documented in 46.9% (90/192) of consultations and for 40.9% (99/242) of problems. The median average documentation of spoken safety-netting advice by GP was 33.3% of problems with a range of 0% to 86.7%. There were five consultations, from four GPs, assessing six problems where no spoken safety-netting advice was observed but was documented in the records. The overall frequency of safety-netting advice observed in records was a third of all consultations (31.9%, 94/295) and a fifth of problems discussed (20.3%, 105/516, Table 3).

**Table 3.** Documentation of problems and spoken safety-netting advice

**Observation (based on verbalisation)**	**Problems discussed**	**Problem documented**		**Verbalised SNA present**		**Documented SNA present**	

	*n*	*n*	%	*n*	%	*n*	%
All problems	516	453	87.8	242	46.9	105	20.3

Acute/AoC problem	315	278	88.3	169	53.7	84	26.7
Chronic problem	201	175	87.1	73	36.3	21	10.4

First presentation (new problem)	102	90	88.2	55	53.9	32	31.4
Not first	392	348	88.8	180	45.9	70	17.9
Unclear	22	15	68.2	7	31.8	3	13.6

Single problem in consultation	156	156	100	99	63.5	53	34.0
Multiple problems in consultation	360	297	82.5	143	39.7	52	14.4

Order assessed by GP (in multi-problem consultations, *n*= 139)							
1	139	128	92.1	70	50.4	25	18.0
2	139	114	82.0	51	36.7	21	15.1
3	60	42	70.0	17	28.3	6	10.0
≥4	22	13	59.1	5	22.7	0	0

*AoC = acute on chronic for example,, acute exacerbation of asthma. SNA = safety-netting advice.*

### Content of spoken and documented safety-netting advice

Table 4 compares how the different components of safety-netting advice were spoken and documented. The mean number of different symptoms/conditions patients were told to look out for, for each problem where safety-netting advice was issued, was 2.2 (SD 1.8, maximum 11) for spoken advice compared with a mean of 1.4 (SD 0.7, maximum 4) for documented advice. No GPs explicitly documented that they had provided written safety-netting advice but in 11 records there was evidence that a patient information leaflet was issued (data not shown).

**Table 4.** Content of safety-netting advice documentation compared with verbalisation for each problem^a^

**Safety-netting advice coding question and codes from observing consultation/medical records**	**Verbalised SNA (*n*= 516)**		**Documented SNA (*n*= 516)**	
	
	***n***	**%**	***n***	**%**
Presence				
Present	242	46.9	105	20.3
Absent	274	53.1	411	79.7

Problem or treatment safety-netting advice				
Problem only	134	26.0	68	13.2
Treatment/management plan only	18	3.5	6	1.2
Both/mixture	90	17.4	31	6.0

Format				
Conditional warning only (for example, ‘worsening advice given’)	2	0.4	3	0.6
Conditional + course of action (for example, if ‘x happens do y’)	240	46.5	102	19.8

Number of different symptoms/conditions to look out for				
1	119	23.1	76	14.7
2	48	9.3	19	3.7
3	32	6.2	9	1.7
4	17	3.3	1	0.2
≥5	23	4.5	0	0.0
Implicit conditional only^b^	3	0.6	0	0.0

Symptom/condition category present				
New specific symptom	82	15.9	22	4.3
Current symptom(s) persists	98	19.0	52	10.1
Worsening/deterioration	45	8.7	18	3.5
Other^c^	66	12.8	9	1.7
Problems/issues	52	10.1	6	1.2
Return of symptoms	23	4.5	6	1.2
Need/as required (PRN)	20	3.9	26	5.0
Concerns/worried/struggling	18	3.5	4	0.8
Changes	12	2.3	1	0.2
Unwell	8	1.6	1	0.2

Generic or specific advice				
Includes specific (for example, cough up blood, chest pain, not better in a set time period)	135	26.2	24	4.7
All generic (problems, issues, concerns, worse, not better [without time course])	107	20.7	81	15.7

Action advised (highest code reported)				
No action (conditional warning only)	2	0.4	3	0.6
Contact other in-hours medical service	5	1.0	0	0.0
Return to practice/same GP	225	43.6	100	19.4
Contact out-of-hours service	4	0.8	0	0.0
Contact emergency services (highest code)	6	1.2	2	0.4

Timescale of action (highest code reported)				
Not specified	175	33.9	72	14.0
Named/fixed time (‘2 weeks’)	48	9.3	15	2.9
Immediate (‘see stat if any change’ – highest code)	19	3.7	18	3.5

a *Verbalised codes obtained from previous study.^14^*

b *Example^‘^So 3 months if not before’.*

c *Other conditions include: develop new ‘symptoms’, ‘want to come back’, ‘not tolerating it’, ‘fed up’, ‘questions’, referral haven’t heard, starts to limit function. Full criteria are listed in the codebook.^21^ SNA = safety-netting advice.*

### Variables associated with documentation of spoken safety-netting advice

Table 5 describes variables that were associated with an altered GP documentation frequency of spoken safety-netting advice. In the univariable analysis, GPs were more likely to document their safety-netting advice for problems that were acute (*P* = 0.005), first presentations (*P* = 0.006), if only one problem was assessed in the consultation (*P* = 0.018), or if the GP had verbalised specific advice (for example, *‘ I’d want you to come back if you start coughing up horrid coloured stuff, greeny-browny, or if you start coughing up any blood’*), rather than generic advice (for example, *‘any problems, let me know’*) (*P* = 0.003). In the multivariable model, the associations between higher documentation and new problems (*P* = 0.030), if only one problem was assessed in the consultation (*P* = 0.040), and specific advice (*P* = 0.007) were maintained, but the association for acute problems was attenuated (*P* = 0.19).

**Table 5.** Variables associated with documentation of spoken safety-netting advice^a^

**Codes from observing consultation/linked data**	**Problems with verbal SNA, *n***	**SNA documentation,**		**Univariable model OR (95% CI) (*n* = 242)**	***P*-value**	**Multivariable model OR (95% CI) (*n* = 235)**	***P*-value**
		***n***	**%**				
Specific SNA verbalised	135	69	51.1	4.22 (1.64 to 10.87)	0.003	3.00 (1.36 to 6.64)	0.007
Only generic SNA verbalised	107	30	28.0				

Acute/acute on chronic problem	169	80	47.3	3.23 (1.43 to 7.33)	0.005	1.70 (0.77 to 3.75)	0.19
Chronic problem	73	19	26.0				

First presentation of problem	55	31	56.4	3.65 (1.44 to 9.21)^b^	0.006	2.69 (1.10 to 6.56)	0.030
Not first	180	65	36.1				
Unclear (exclude)	7	–					

Single problem in consultation	99	50	50.5	2.75 (1.19 to 6.34)	0.018	2.17 (1.04 to 4.55)	0.040
Multiple problems in consultation	143	49	34.3				

Planned follow-up documented	142	52	36.6	0.56 (0.28 to 1.15)	0.11	0.63 (0.33 to 1.20)	0.16
No planned follow-up documented	100	47	47.0				

GP aged <50 years	178	80	44.9	2.59 (0.79 to 8.54)	0.12	2.06 (0.70 to 6.04)	0.19
GP aged ≥50 years	64	19	29.7				

a *Both models adjust for clusters of problems seen by the same GP and problems raised by same patient. Multivariable model includes all variables in table as covariants.*

b *Excludes seven consultations with unclear first presentation code (n = 235). CI = confidence interval. OR = odds ratio. SNA = safety-netting advice.*

### Comparison between verbalised and documented models

Table 6 shows two models with ORs for variables associated with a higher or lower frequency of verbalised or documented safety-netting advice from 274 consultations. Although the frequency of documented safety-netting advice in the medical records was lower than what was spoken, the associations that verbalised safety-netting advice was more likely to be present for acute problems (*P* = 0.004), when only one problem was discussed during the consultation (*P* = 0.001), and problems assessed by the GPs aged <50 years (*P*<0.001) were also found when medical records instead of verbatim transcripts were analysed (*P* = 0.001, *P* = 0.032, *P* = 0.028, respectively).

**Table 6.** Comparison of models based on verbalised and documented safety-netting advice^a^

**Codes from observing consultation/linked data**	**Verbalised problems (*n*= 465)**			**Documented problems (*n*= 415)**		
	
	**SNA present, %**	**OR (95% CI)**	***P*-value**	**SNA present, %**	**OR (95% CI)**	***P*-value**
Acute/AoC problem	52.8	2.14 (1.27 to 3.62)	0.004	30.2	3.91 (1.78 to 8.61)	0.001
Chronic problem	36.1			11.0		

First presentation	53.1	0.97 (0.54 to 1.76)	0.92	35.6	1.97 (0.93 to 4.20)	0.078
Not first	44.4			19.2		

Single problem in consultation^b^	61.8	2.58 (1.51 to 4.43)	0.001	30.9	2.11 (1.07 to 4.18)	0.032
Multiple problems in consultation	39.3			17.4		

Follow-up present^b^	43.8	0.69 (0.42 to 1.14)	0.15	19.1	0.42 (0.22 to 0.80)	0.009
No follow-up	51.7			29.1		

GP aged <50 years	53.9	2.88 (1.60 to 5.16)	<0.001	29.0	3.77 (1.16 to 12.30)	0.028
GP aged ≥50 years	32.7			11.8		

Patient age ≥65 years	47.0	1.23 (0.68 to 2.22)	0.50	22.7	1.30 (0.61 to 2.74)	0.50
Patient age 18–64 years	45.9			22.6		

Patient sex: female	46.3	0.84 (0.50 to 1.40)	0.50	22.7	1.20 (0.61 to 2.36)	0.59
Patient sex: male	46.1			22.6		

Patient ethnicity: other	54.8	1.15 (0.47 to 2.83)	0.76	36.4	1.18 (0.35 to 3.95)	0.79
Patient ethnicity: white	45.4			21.5		

Index of Multiple Deprivation quintile	Wald test *P* = 0.59			Wald test *P* = 0.57			

a *Data from 274 consultations. Multivariable multilevel mixed-effects modelling to adjust for problems seen by the same GP and multiple problems raised by the same patient. Problems with missing data were excluded.*

b *Codes assessed separately based on model type, for example, if two problems discussed but only one documented, coded as single problem consultation in documentation model. AoC = acute on chronic. CI = confidence interval. OR = odds ratios. SNA = safety-netting advice.*

### Documentation is influenced by the order that problems are assessed in multiproblem consultations

There were 139 consultations where multiple problems were discussed (range 2–7 problems). In consultations for multiple problems, the later a problem was discussed, the less likely GPs were to document the problem (OR 0.50 per unit increase, 95% CI = 0.37 to 0.67, *P*<0.001 adjusted for if problems were acute or first presentations as covariates, *n* = 136 consultations, 342 problems; see Table 3 for unadjusted frequencies).

The frequency of spoken and documented safety-netting advice in consultations with more than one problem also decreased the later a problem was assessed by the GP (OR 0.68 per unit increase, 95% CI = 0.50 to 0.92, *P* = 0.011 and OR 0.57 per unit increase, 95% CI = 0.36 to 0.92, *P* = 0.022, respectively, adjusted for all covariates in Table 6 verbalised model, *n* = 129 consultations, 324 problems).

## DISCUSSION

### Summary

There was substantial variation in how often GPs documented the safety-netting advice they had given to patients, which ranged from no documentation to almost nine out of every 10 problems.

GPs were more likely to document their spoken safety-netting advice when assessing new problems, when they had verbalised specific rather than generic safety-netting advice, and when only one problem was assessed in the consultation. In consultations where more than one problem was discussed, the later a problem was assessed, the less likely there was to be spoken or documented safety-netting advice.

### Strengths and limitations

To the authors’ knowledge, this is the first study to describe in detail how safety-netting advice is recorded in medical records compared with directly observed spoken advice during GP consultations. The GPs in the archive knew they were being recorded, although they did not specifically know their safety-netting practices would be assessed, minimising potential ‘Hawthorne effects’.^24^ The exact impact of recording consultations for research purposes on GP behaviours is complex.^25^ It is conceivable that the findings of this current study may represent GPs attempts at ‘best practice’ and hence overestimate the consistency of routine safety-netting practices. Similarly, it is feasible that there may be unmeasured characteristics more common to clinicians who self-selected to be video-recorded for the archive, such as confidence in their standard of practice, which may again suggest the current findings would overestimate the consistency of practice in the real world.

This was a secondary analysis of a pre-existing dataset of face-to-face, adult patient, routine UK GP consultations only; the sample size was fixed and not generated based on a power calculation. The small sample size and the lack of representativeness of this sample — 295 adult consultations (87.5% self-reported white ethnicity) with 23 GPs (all white ethnicity) from 12 practices in the West of England — may reduce the generalisability of these findings to other settings. Indeed, even in this small sample the authors observed large variation between GPs. The consultations in the archive were recorded in 2014–2015 and contemporary practice may have changed. Finally, because of the cross-sectional nature of the study design, it was not possible to tell if patients had previously been given safety-netting advice for the same problem.

### Comparison with existing literature

The GPs in this study often failed to document safety-netting advice, and were less consistent at doing so than primary and secondary care healthcare professionals in studies measuring safety-netting when managing feverish children sent home.^7^ This is not unexpected owing to the potentially serious nature of feverish illnesses in children and specific guidance that safety-netting advice should be given.^26^

GPs in the current study were more likely to document specific safety-netting advice when given, which may be more pertinent to patients as the usefulness of generic safety-netting advice has been questioned from a patient’s perspective.^27^

This study reports that in under half of problems (99/242) where safety-netting advice was given it was also recorded in the medical records. This is 10 fewer problems than reported in the previous study undertaken by the same group.^14^ In the current study, coders assessed medical records in isolation and did not take into account what was verbalised. For example, one GP verbalised *‘So if you’re getting indigestion pains, coughing up blood, or your stool is very dark and black and sticky, you must stop the naproxen and come and see me straight away’* but only documented ‘discussed possible S/Es [side effects]’. In this current study, these episodes were not coded as documented evidence.

The UK is reported to have an average consultation duration lower than many economically comparable countries.^28^ It has been reported that on average, GP consultations contain 2.5 problems and only increase by 2 min for each additional problem raised.^17^ This may not be sufficient time to comprehensively assess, safety-net, and document all problems. The findings in this study suggest patterns of prioritisation in documentation of both clinical problems and safety-netting advice that may be a response to such time pressures.

### Implications for research and practice

The finding in this study that over half of safety-netting advice for problems raised in routine GP consultations goes undocumented highlights that retrospective reviews of medical records^16^ are likely to under-report the frequency of safety-netting advice given in primary care.

Biases in GP documentation practices such as being more likely to document for new problems, when only a single problem was discussed, and when specific safety-netting advice is given (Table 5) may also have an impact. However, as similar associations were found between altered frequencies of safety-netting advice and key variables (Table 6) when comparing spoken with documented advice, large studies of medical records are still likely to be a good platform for researching safety-netting behaviours but should be interpreted with caution. Medical records have the advantage of being routinely collected, and large anonymised datasets for research purposes are easier to create and access than comparable datasets of video-/audio-recorded consultations.

The medical notes edition of the coding tool (SaNCoT) used in this current study was much quicker to use and had a higher level of coder agreement than the more complex observational coding of recorded consultations (κ = 0.87 versus κ = 0.66).^3^ As such, it is likely to have greater utility in everyday GP work to audit local clinical practice and is available freely.^21^ The fastest and least time-consuming method would be an automated search of documented safety-netting advice.

Automated searches remain limited at present as most advice is currently recorded as free text and not coded. However, the use of safety-netting templates, with searchable codes may in part address this and is under evaluation.^29^ Such searches could inform interventions seeking to identify and minimise unwarranted variation in practice.^30^

With the rise of telephone and e-consulting because of the COVID-19 pandemic, telephone texting systems with pre-defined templates that automatically insert and code into medical records may offer an avenue for improving documentation and patient access to written advice, for which there is patient demand.^7^^,^^31^ Those with low literacy skills have voiced a preference for an audio–visual format of safety-netting,^32^ which lends itself to smart phone messaging. Texting patients safety-netting advice has been found to be acceptable to GPs but more patient-focused research is needed.^33^ However, adopting this into routine practice for all patients may contribute to the inverse care law,^34^ where those without access to a working mobile/smart phone or with health literacy issues could receive a lower quality of care.

This study, and others,^15^^,^^17^ evidence a common disparity between what is said and what is documented in primary care consultations. This potentially leaves those GPs whose documentation is incomplete vulnerable to challenge regarding their practice. Routine audio-recording of all consultations offers one objective avenue for resolving disputes based on this incongruity and is already occurring for many telephone encounters.

Although recording has not been widely incorporated into face-to-face consultations, some patients are already openly and covertly recording healthcare encounters,^35^ which are admissible evidence in court.^36^ Despite existing precedents, recording consultations would require clinician and public support and should aim at reducing GP administration time.

Recent estimates have suggested a ‘substantial burden of avoidable significant harm’ in English primary care, mostly attributable to diagnostic error, medication incidents, and delayed referrals.^37^ Such findings and the study of patient safety incident reports emphasise how effective safety-netting advice and its consistent documentation may help to minimise patient harm.^38^

The observation, in this study, that GPs are less likely to verbalise safety-netting advice when more than one problem is assessed in a consultation, and they are less likely to document safety-netting advice they have given, should prompt GPs to consider how safely they can assess and document more than one problem in a single consultation, and this risk should be shared with patients to help manage expectations.
